# Criterion-Related Validity of a Simple Muscle Strength Test to Assess Whole Body Muscle Strength in Chinese Children Aged 10 to 12 Years

**DOI:** 10.1155/2018/2802803

**Published:** 2018-01-18

**Authors:** Liqin Yin, Changfa Tang, Xia Tao

**Affiliations:** ^1^Department of Physical Education, Hunan Normal University, Lusan North Road, Changsha, Hunan 410082, China; ^2^Department of Physical Education, Hunan University of Technology, Taishan Road, Zhuzhou, Hunan 412008, China; ^3^Department of Physical Education, Hunan Business College, XiaJiaHu Road, Changsha, Hunan 410089, China

## Abstract

**Objective:**

To study the criterion-related validity of simple muscle strength test (SMST) indicators and assess whole body muscle strength in Chinese children aged 10 to 12 years old.

**Methods:**

Two hundred and forty children were equally divided into four groups in different genders and residences. The SMST indicators (hand-grip, knee bent push-up, back muscle strength, sit-up, leg muscle strength, and standing long jump) were tested. We set up the total level of the whole-body muscle strength (*F*_total_) through testing isokinetic muscle strength of the six joints' flexion and extension movements. Pearson correlation analyses were used to analyze the correlation between the SMST indicators and the *F*_total_.

**Results:**

(1) Leg muscle strength and back muscle strength demonstrated the highest validity scores. Sit-ups, hand grip, and standing long jump demonstrated the lowest validity scores. (2) Leg muscle strength had the highest validity for males, but back muscle strength had the highest validity for females.

**Conclusions:**

Back muscle strength and leg muscle strength can give the highest validity of assessing whole body muscle strength, and also has higher validity in both the urban and rural children. For urban children, but not rural, the knee bent push-up also has a high validity indicator.

## 1. Introduction

The effectiveness of strength-related quality in daily life, exercise, and prevention and treatment of chronic diseases has increasingly been recognized [1, 2]. As a result, strength quality is included in the physical fitness tests as an essential test in many regions, worldwide [3, 4]. Strength tests are an important component of physical fitness tests in many countries and regions in the current world. The simple muscle strength test (SMST), an important strength quality test, typically uses simple force, range, and speed measuring devices to measure the strength of muscles in static and dynamic states. These low-cost and portable devices are an appropriate and convenient method to assess muscle strength during physical fitness tests. Fan et al. [5] reviewed eleven physical testing systems across seven countries/regions and found that there were eighteen indicators of SMST. The most commonly used SMST indicators of physical fitness tests in Chinese children aged 10–12 years are hand-grip, knee bent pull-up, back muscle strength, leg muscle strength, standing long jump, and sit-up.

Ample research has been conducted on the criterion-related validity of SMST test indicators [6–10]. The criterion standard for various tests (1RM test [11], isokinetic muscle test [12], force plates [13], and surface EMG contribution rate [5]) is usually adopted. The validity of the upper limb SMST indicators is more prominent for modified pull-ups, hand-grip, standard push-ups, and bent-knee push-ups [14]. There were three indicators in the test of trunk strength: sit-ups, trunk-lift, and back muscle strength. Standing long jump and leg press are common indicators of lower-body muscle strength; however, research suggests that the leg press is not suitable for large-scale crowd testing [7]. The standing long jump is a basic test of young children's lower-body strength and is favorable for its utility, time-saving, and economy of use [4]. However, literature [15] suggests that the standing long jump has questionable validity against technology, anthropometry, biomechanics, and coordination control factors.

However, there are two main defects in previous studies. Firstly, there were no tests for two or more than two blocks and no single test can assess the overall strength of the body. It is necessary to study the validity of the SMST to reflect the whole body muscle strength. Secondly, residence differences are not considered in the study of validity. Fan et al. [5] further suggested that the indicators of SMST were not unified across countries/regions [16]. The indicator of the same position was different across age and gender. Additionally, there is a place of residency difference in muscle strength tests [17]. The difference of muscle strength of urban and rural adolescents is more obvious [18]. Although gender differences are reflected in the indicators of strength testing, there is less attention to the differences in urban and rural areas.

The aims of the present study, therefore, were to (1) study the criterion-related validity of SMST indicators (hand-grip, knee bent pull-up, back muscle strength, leg muscle strength, standing long jump, and sit-up) to assess whole body muscle strength in Chinese children, aged 10 to 12 years and (2) compare the validity of SMST indicators to assess whole body muscle strength in different genders and residences.

In order to study the validity of SMST indicators to assess whole body muscle strength, we tested the isokinetic muscle strength of the six joints' flexion and extension movements of the whole body. The isokinetic strength test (IST) has been demonstrated to be valid for assessing muscle strength and muscle function in the knee joint [19, 20], hip joint [21, 22], ankle joint [23], shoulder joint [24], and trunk [25, 26]. The public factors were extracted by conducting a factor analysis and the total factor score was calculated (*F*_total_) according to the public factor contribution rate [27]. We set up the total level of the whole body muscle strength (*F*_total_) and used it as the calibration of whole body muscle strength. Meanwhile, we analyzed the SMST indicators and the *F*_total_, and screened out the best index reflecting the whole body muscle strength of children. Pearson correlation analysis was carried out between the SMST indicators and the whole body strength (*F*_total_) to screen out the best indicator to reflect the muscle strength of the children.

## 2. Methods

### 2.1. Participants and Design

Fifth and sixth graders, aged 10 to 12 years, were recruited from the Hunan Normal University Affiliated Primary School (Changsha City, urban) and Liuyang Kouchong Primary School (Liuyang, rural) using a stratified random sampling method. In total, 240 students participated in the study and were divided into four groups which were urban boys (UB), urban girls (UG), rural boys (RB), and rural girls (RG). Each group contained 60 students. All participants met the following criteria: (1) aged 10 to 12 years; (2) no history of disease and contraindications; (3) did not participate in high-intensity exercise in the seven days before the test; (4) had no joint, muscle, or bone damage in the first half of the year before the test; (5) carried out adequate preparation activities before the test.

### 2.2. Measurements

#### 2.2.1. Isokinetic Strength Test (IST)

The Biodex system 3 is a multijoint, constant velocity testing and training system (Biodex Medical Systems New York, USA) and was used to assess IST in the present study. The test indicators for IST were based on the six joints (shoulder, elbow, wrist, marrow, knee, and ankle) for peak torque (PT) and unit weight torque (UWT). PT is the maximum torque output produced by muscle contraction throughout the joint activity, that is, the highest point of the torque curve [28]. UWT refers to the peak torque of unit weight and can be used to compare the strength of the individual people with different weight [22, 23].

Range of motion (ROM), also known as the range of joint activity, refers to the maximum radian that can be achieved during joint activity [24]. There were three types of ROM of the joints, which were 60°/s, 180°/s, and 240°/s, respectively, standing for slow, medium, and fast test speeds. Due to incomplete muscle development of participants and because the participants could not complete the 60°/s test, the current study used the 90°/s test. The full range was adopted for motion amplitude. The IST was strictly conducted following the instruction in the manual of Biodex system 3. For each joint, there were three groups of tests and each test was tested three times with 30-second periods of rest between each test. Before the formal test, the submaximal joint flexion and extension exercises were performed to help participants familiarize themselves and adapt to the test. Afterwards, the participants were weighed to correct the effect of gravity on the test results. The sagittal axis of the joint tested was aligned with the axis of the spindle of test equipment. The order of the six joints tested was as follows: hip; shoulder; knee; elbow; ankle; wrist. The upper and lower limb tests were carried out alternately.

#### 2.2.2. Simple Muscle Strength Test (SMST)

The introduction of SMST is presented in Table 1. Each test was measured thrice, with 3 to 5 minutes of rest between each test. The best result was recorded.

### 2.3. Statistical Analyses

#### 2.3.1. The Establishment of *F*_total_ Based on IST

*F*_total_ reflects the overall level of muscle strength of the human body. It was used as a standard of comparison to evaluate the validity of the muscle strength test. The *F*_total_ uses a few factors to describe the indicators and the weight of the link between various factors and was closely related to several variables as the same factor, with fewer factors reflecting the original data of general information—establishing a test for muscle strength evaluation and the effectiveness of the standard of comparison. In the current study, *F*_total_ was counted through factor analysis of the 90°/s PT of IST.

Before conducting the factor analysis, the correlation analyses, Kaiser-Meyer-Olkin (KMO) test and Bartlett test, were used to test whether the data met the criteria for conducting a factor analysis. The Bartlett's sphericity test showed whether the data was suitable for factor analysis. According to the principle of factor analysis, when the cumulative contribution rate of extracted common factor was over 80%, this factor could explain most of the information [27]. We then extracted the public factors by conducting a factor analysis and calculated the total factor score (*F*_total_) according to the public factor contribution rate.

#### 2.3.2. Data Analysis

To study the validity of the SMST indicators, Pearson correlation analyses were used to analyze the correlation of the SMST indicators and the *F*_total_. Two-way analysis of variance (residence *∗* gender) was used to investigate the differences in the indicators of SMST. All statistical analyses were performed using the Statistical Package for Social Sciences (SPSS, v.16.0; SPSS Inc., Chicago), and the level of significance was set at 0.05.

## 3. Results

### 3.1. Participant Anthropometry

As shown in Table 2, the height of urban students was significantly higher than that of rural students (*p* < 0.05), especially among girls (*p* < 0.05). In terms of weight, urban students were significantly heavier than rural students (*p* < 0.05). In terms of vital capacity, boys had significantly higher scores than girls (*p* < 0.05), but the difference was not significant across place of residence.

### 3.2. Simple Muscle Strength Test

As shown in Table 3, the upper limbs of urban students scored significantly higher than rural students (*p* < 0.05). Boys scored significantly higher in the kneeling push-up than girls (*p* < 0.01). In addition, urban children scored significantly higher in the kneeling push-up than rural children (*p* < 0.05). Back muscle strength was greater in the male than in the female participants (*p* < 0.01); rural children had greater back muscle strength than urban children (all *p* < 0.01). Specifically, rural boys scored higher in the back muscle strength test than urban girls. There was no significant difference in sit-up across gender and place of residence. Boys had significantly greater leg muscle strength than girls (*p* < 0.01) while rural participants scored higher in leg muscle strength test than urban participants, especially among boys (*p* < 0.01). Boys scored higher in the standing long jump than girls, with rural boys scoring the highest and urban girls the lowest. There was no difference in the standing long jump between urban and rural children.

### 3.3. Isokinetic Strength Test

#### 3.3.1. Peak Torque of Six Joints

The results of IST are presented in Table 4. Compared with the right shoulder, the 90°/s, 180°/s, and 240°/s PT of the left shoulder flexor were significantly lower (all *p* < 0.01). Compared with the right hip, the extended 180°/s PT and flexor 90°/s and 180°/s PT of left hip were significantly higher (all *p* < 0.01). The 240°/s PT of left hip flexor was significantly higher than that of the right hip (*p* < 0.05).

Compared with the right knee, the 90°/s PT (*p* < 0.01) and 180°/s PT (*p* < 0.05) of left knee flexor were significantly lower. Compared with the right ankle, the 90°/s, 180°/s, and 240°/s PT of left ankle extensor were significantly higher (all *p* < 0.01) while the 90°/s PT (*p* < 0.01) and 180°/s PT (*p* < 0.05) of left ankle flexor were significantly lower. For the other joints, the PTs of right side were higher than the left side, but the differences were not significant (*p* > 0.05).

#### 3.3.2. Unit Weight Torques of Six Joints

The results of unit weight torque (UWT) are similar to those of PT (Table 5). Compared with the right shoulder, the 90°/s, 180°/s, and 240°/s UWT of left shoulder flexor were significantly lower (all *p* < 0.01). Specifically, the 180°/s, 240°/s, and 90°/s UWT of extensors and 90°/s UWT of flexors under working conditions were significantly different; the left shoulder is lower than the right shoulder (all *p* < 0.01). There were also differences in 90°/s UWT of extensor and 180°/s and 240°/s UWT of flexor between left and right shoulders: left were lower (all *p* < 0.05). Compared with the right hip, the 90°/s, 180°/s, and 240°/s UWT of left hip flexors were higher (*p* < 0.01).

Compared with the right knee, the 90°/s UWT of left knee flexor under working condition was significantly lower (*p* < 0.05). Compared with the right ankle, the 90°/s, 180°/s, and 240°/s UWT of left ankle extensor were significantly higher (all *p* < 0.01). The 90°/s, 180°/s, and 240°/s UWT of left ankle flexor of were significantly lower (all *p* < 0.01). In total, the UWTs of other joints of the right side were higher than those of the left side, but the difference was not significant.

### 3.4. Validity of SMST Indicators

#### 3.4.1. Correlations between Indicators of SMST

The correlation coefficients between indictors of SMST are presented in Table 6. Hand-grip strength was significantly correlated with back and leg muscle strength (all *p* < 0.05); kneeling push-up was significantly related to hand-grip strength and back and leg muscle strength (all *p* < 0.05); back muscle strength was significantly correlated with leg muscle strength (*p* < 0.05). Meanwhile, the correlations between sit-ups and the standing long jump with the remaining indicators of SMST were not significant (*p* > 0.05). (Table 6).

#### 3.4.2. Criterion-Related Validity of SMST Indicators to Access Whole Body Muscle Strength

**Figure 1:**
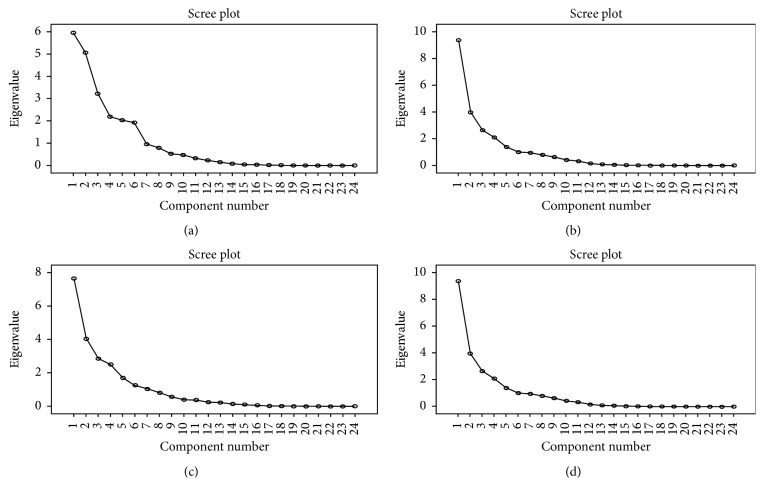
Common factor and feature scatter plot. (a) Urban boys, common factors = 7; (b) urban girls, common factors = 6; (c) rural boys, common factors = 5; (d) rural girls, common factors = 5.

The KMO and Bartlett test results showed that all correlation coefficients between each indicator of IST were greater than 0.3 for UB, UG, rural boys, and RG. The KMO test results for UB, UG, RB, and RG were, respectively, 0.876, 0.833, 0.856, and 0.872. Bartlett's sphericity test showed that the data was suitable for factor analysis (*p* < 0.001). According to the principle of factor analysis, when the cumulative contribution rate of extracted common factor was over 80%, this factor can explain most of the information (Figure 1) [31].

There were seven common factors for urban boys (Table 7), with the cumulative contribution rate of 83.67%. There were six common factors for urban girls (Table 8), with a cumulative contribution rate of 80.64%. There were five common factors for rural boys (Table 9), with a cumulative contribution rate of 83.26%. For rural girls, there were five common factors (Table 10), with a cumulative contribution rate of 81.46%. The total score (*F*_total_) was calculated according to the contribution rate of each common factor.

The correlation coefficients were shown in Table 11. For UG, the correlation coefficient between *F*_total_ and back muscle strength was the highest (*r* = 0.803, *p* < 0.01), while the correlation coefficients between *F*_total_ and leg muscle strength were the highest among UB (*r* = 0.811, *p* < 0.01), RB (*r* = 0.837, *p* < 0.01), and RG (*r* = 0.801, *p* < 0.01), followed by the correlation coefficients between *F*_total_ and back muscle strength (urban boys: *r* = 0.774, rural boys: *r* = 0.824; rural girls: *r* = 0.799, all *p* < 0.01). The correlation coefficients between *F*_total_ and hand-grip were the lowest among UB (*r* = 0.611, *p* < 0.01), RB (*r* = 0.635, *p* < 0.01), and RG (*r* = 0.548, *p* < 0.01).

## 4. Discussion

The main purpose of this study was to determine the criterion-related validity of the SMST indicators for hand-grip strength, knee bent pull-up, back muscle strength, leg muscle strength, standing long jump, and sit-up to assess whole body muscle strength in Chinese children aged 10 to 12 years. Gender and residence were included as between-subject factors because earlier studies have shown differences in the concurrent validity between males and females and urban and rural populations in muscle strength [32, 33]

The results showed that the criterion-related validity of SMST indicators to assess whole body muscle strength was moderate to high. The correlation SMST and *F*_total_ ranged from 0.425 to 0.811. In addition, leg muscle strength and back muscle strength demonstrated the highest validity scores, *r* = 0.811 and 0.80, respectively; sit-ups, hand-grip, and standing long jump demonstrated the lowest validity scores, *r* = 0.425, 0.455, and 0.465, respectively. In the upper body, knee bent push-up demonstrated greater validity than hand-grip strength; in the trunk, back muscle strength is more valid than sit-ups; in the lower body, leg muscle strength is more valid than the standing long jump.

Similar results were found in several other studies. For instance, in Japan, back, hand-grip, wrist, leg, and abdominal muscle strength were adopted as indicators [34]. The validity of the indicators was examined using the mean and standard deviation of each test indicator as the standard of comparison. The results showed that the correlation coefficient between back muscle strength and the standard of comparison (*r* = 0.9172) was higher than hand-grip and leg strength. Prior research has examined the validity of indicators of the upper limb strength quality tests. The research has examined the convergent validity of push-ups, pull-ups, hand-grip, and the inverted row. They found that the convergent validity of the push-up was the highest among all indicators while the rest of the indicators were not sufficiently valid. The results showed that hand-grip strength was a validity indicator [35, 36]. However, some studies also found that the hand-grip test was not valid [37], especially in accessing whole body muscle strength. Previous studies suggested that the knee bent push-up was a valid indicator of upper limb strength quality test [38, 39]. Researchers used the 1 repetition maximum (1RM) leg extension test to assess the validity for lower-body muscular power and found that the standing long jump test can be a useful tool to assess lower-body muscular strength in children, but not whole body muscle strength [40]. Children between the ages of 6 and 17 were tested [41]. The lower limb strength test included standing long jump, vertical jump, squat jump, and the countermovement jump. Using a multivariate regression analysis, this study analyzed the relationship between indicators of upper limb strength tests. In assessing whole body muscle strength, Tillin et al. [42] recommended multijoint rather than single joint testing due to the specific neural and mechanical conditions in athletic performance tasks, such as sprinting and jumping. The results showed that the correlation coefficients between the standing long jump and other indicators of upper limb strength tests were low [43]. However, the standing long jump has also been challenged by researchers because the test results were susceptible to factors, such as skill learning. This indicated that back muscle strength and leg muscle strength may give the highest validity of assessment for whole body muscle strength.

The present study also sought to compare the validity of SMST indicators to assess whole body muscle strength in different genders and residences. The results for back muscle strength demonstrated the highest validity scores for UB, RB, and RG, *r* = 0.811, 0.837, and 0.801, respectively, and leg muscle strength demonstrated the highest validity scores for UG, *r* = 0.803. Leg muscle strength demonstrated the highest validity for males, but this is only the case for back muscle strength for females. Back muscle strength and leg muscle strength have higher validity in both the urban and rural children. For urban children, the knee bent push-up also has a high validity indicator but has poor validity for rural children.

There were differences in anthropometric characteristics between urban and rural children, and also differences in the SMST results. The evaluation of physical quality fitness tests for students has been concentrated on the adaptability of the test among children of different ages and genders around the world. Generally speaking, there were three kinds of situations in terms of the difference of indicators across age and gender. First, the indicators of test for junior students were same across gender, while the indicator for senior students was different from that of junior students, and there were differences in indicators between male and female senior students. For instance, in the School Physical Fitness Rewards Program of Hong Kong [44], general hand-grip strength was the indicator of muscle strength of upper limb for primary school students. Push-ups and modified push-ups were the indicators of muscle strength of the upper limbs for male and female middle school students. Second, the indicator of senior students' genders was the same as that of junior students, while the indicator of senior students of the other genders was modified. For example, in the National Physical Fitness Award Program of Singapore [45], the indicator of muscle strength of the upper limbs was the modified pull-up for both males and females younger than 15 years. For those older than 15 years, the indicator for female students remained the modified pull-up while that of male students was the pull-up. Third, the indicator was different between male and female junior students. In addition, the indicator has also changed for senior students. For instance, the national education standards of the course “Sports” in Russia [46–48] stated that the indicator for girls aged 7 to 15 years was the overhanging arm flexion and extension, while that for girls aged 16 to 17 years was bicep curl to pull-up.

## 5. Conclusion

Back muscle strength and leg muscle strength can give the highest validity for assessing whole body muscle strength and also have higher validity in both urban and rural children. For urban children, the knee bent push-up also has a high validity indicator but poor validity for rural children.

In the future, back muscle strength and leg muscle strength can be considered as strength test indicators for Chinese student's physical fitness test. Meanwhile, residential differences in SMST indicators should be considered when setting up the physical fitness test evaluation indicators of students, especially for knee bent push-up.

## Figures and Tables

**Table 1:** Indicators and test methods of the SMST.

Test site	Indicators	Test methods	Instruments
Upper limbs	Hand-grip (kg) [29]	The participant stood, relaxed, with his/her arms naturally drooped to the sides of the body, palms inward, the hand to be tested holding the grip dynamometer, holding the strength measuring site, reaching slightly out. The range did not exceed 30°. When the participant was ready, he/she was required to use their maximum strength to grip the grip dynamometer once and read the record. Left and right hands were measured alternately.	The Hui Hai student physical quality evaluation system (Hui Hai Electronics, Shanghai, China)
	Knee bent push-up (times) [30]	The participant began prone on the mat, with ten fingers forward and shoulder-width apart, on the ground. The arms were straight and the knees were on the mat. The feet were off the ground and overlapped each other and maintained a straight line from the head and the back to the knee. After hearing the signal, the participant flexed his/her arms to about 90° before pushing the body up and straightening his/her arms. This was counted as a push-up. Throughout the test, the participant held a straight line from the head and back to the knee, and there was no obvious pause between the movements of kneeing push-up. It took 1 minute to complete the knee bent push-up.	The Hui Hai student physical quality evaluation system (Hui Hai Electronics, Shanghai, China)

Trunk	Back muscle strength (kg) [30]	The participants stood and adjusted the handle height of the back dynamometer to make the upper limb of the participant lean forward at 30°. During the test, the participants had both hands clenched on the handle and kept their legs straight, using the maximum strength of the arm, pulling up the back dynamometer. After pulling up the back dynamometer, the participants could not bend their arms or knees, or fall backwards.	BCS-400 electronic back dynamometer (Hengqi Weighing Factory, Nantong, Jiangsu, China)
	Sit-up (times) [29]	The participant lied on his/her back on the mat with knees bent, with his/her fingers grasping the head (back of the head) in the ready posture. When the time started, the participant sat up as quickly as possible, with two elbows touching the outer sides of the two legs. Both shoulders, back, and head must be clenched to the mat in the ready posture. It took 1-minute to complete the sit-up.	The Hui Hai student physical quality evaluation system (Hui Hai Electronics, Shanghai, China)

Lower limbs	Standing long jump (m) [29]	The participant stood behind the jump line, with feet naturally apart. The toes should not step on the line (preferably using a rope as the jump line). Both feet jump simultaneously from the standing point; taking a step or successive jumping was not allowed. The vertical distance from the trailing edge of jump line to the nearest landing point was measured.	The Hui Hai student physical quality evaluation system (Hui Hai Electronics, Shanghai, China)
	Leg muscle strength (kg) [29]	The leg muscle strength test also uses the back dynamometer, but the test method is different. The participant handle height of the back dynamometer was adjusted to make the participant bend the knee at ~115°–125°. During the test, the participants had both hands clenched on the handle, holding the upper body upright and using their maximum strength of the arm to pull the back dynamometer. When pulling up the back dynamometer, the participants could not bend their arms, bend their body, or fall backwards.	BCS-400 electronic back dynamometer (Hengqi Weighing Factory, Nantong, Jiangsu, China)

**Table 2:** The anthropometry characteristics of participants.

	UB	RB	UG	RG
Height (cm)	144.64 ± 4.81^▵☆^	140.2 ± 4.05	142.17 ± 9.55^☆^	139.93 ± 6.78
Weight (kg)	40.67 ± 7.41^▵☆^	35.2 ± 8.02	35.98 ± 9.73	33.16 ± 6.07
Vital capacity (ml)	1750 ± 296.24^☆^	1695.9 ± 273.15	1609.5 ± 477.06^☆^	1669 ± 307.01

*Note*. UB: urban boys; RB rural boys; UG: urban girls; RG: rural girls. ^☆^Comparison between children with same place of residence but different gender, *p* < 0.05. ^▵^Comparison between children of same gender but different place of residence, *p* < 0.05.

**Table 3:** The results of SMST.

	UB	RB	UG	RG
Hand-grip (kg)	14.36 ± 2.04^△☆^	11.22 ± 3.79^☆^	12.75 ± 4.10	10.24 ± 3.25
Knee bent push-ups (kg)	16.32 ± 3.27^△☆☆^	14.57 ± 3.78^△☆☆^	11.16 ± 3.49	9.87 ± 2.32
Back muscle strength (kg)	43.4 ± 7.89^△☆^	55.5 ± 11.77^△☆☆^	39.2 ± 9.78	42.8 ± 8.13
Sit-ups (times)	32.14 ± 8.22	33.32 ± 7.56	32.76 ± 7.87	31.59 ± 9.05
Leg muscle strength (kg)	49.9 ± 9.23^△△^	83.8 ± 19.46^△△☆^	45.5 ± 16.07^☆^	55.3 ± 15.22
Standing long jump (m)	1.67 ± 0.45	1.72 ± 0.23^☆☆^	1.53 ± 0.34	1.46 ± 0.49

*Note*. UB: urban boys; RB rural boys; UG: urban girls; RG: rural girls. ^☆^Comparison between children with same place of residence but different gender, *p* < 0.05, ^☆☆^*p* < 0.01. ^△^Comparison between children of same gender but different place of residence, *p* < 0.05, ^△△^*p* < 0.01.

**Table 4:** The peak torques of six joints.

	Extensor PT			Flexor PT		
	90°/S	180°/s	240°/s	90°/S	180°/s	240°/s
Left shoulder	12.97 ± 5.08	8.78 ± 4.95	5.54 ± 4.44	12.02 ± 4.33^*∗∗*^	6.76 ± 6.25^*∗∗*^	1.82 ± 4.6^*∗∗*^
Right shoulder	14.51 ± 6.25	10.61 ± 5.95	8.01 ± 6.48	14.39 ± 6.81	10.23 ± 7.31	5.11 ± 6.53
Left elbow	7.33 ± 3.30	5.97 ± 3.42	4.56 ± 2.73	7.65 ± 2.70	5.13 ± 2.76	2.89 ± 3.04
Right elbow	8.08 ± 3.27	5.76 ± 2.81	4.95 ± 3.07	9.28 ± 2.86	6.98 ± 2.68	4.97 ± 3.21
Left wrist	2.56 ± 1.60	1.18 ± 2.28	0.41 ± 1.08	2.67 ± 1.78	1.82 ± 1.84	1.13 ± 1.40
Right wrist	2.68 ± 3.85	1.02 ± 1.33	0.35 ± 0.91	3.16 ± 1.88	2.14 ± 1.80	1.91 ± 1.77
Left hip	30.8 ± 11.64	26.8 ± 12.32^*∗∗*^	19.9 ± 13.11	39.86 ± 10.08^*∗∗*^	34.42 ± 11.81^*∗∗*^	29.93 ± 12.18^*∗*^
Right hip	26.0 ± 10.76	22.0 ± 11.88	16.0 ± 10.67	35.32 ± 10.30	28.41 ± 10.46	24.06 ± 10.93
Left knee	46.1 ± 12.29	37.3 ± 10.05	32.39 ± 9.23	18.57 ± 7.31^*∗∗*^	18.71 ± 8.00^*∗*^	16.62 ± 7.47
Right knee	47.9 ± 12.86	39.1 ± 10.76	33.37 ± 9.69	23.01 ± 7.40	21.41 ± 7.59	18.16 ± 6.35
Left ankle	23.76 ± 6.54^*∗∗*^	21.00 ± 5.23^*∗∗*^	19.15 ± 5.47^*∗∗*^	3.12 ± 2.58^*∗∗*^	0.96 ± 1.19	0.19 ± 0.34^*∗∗*^
Right ankle	8.38 ± 3.01	5.06 ± 2.41	1.11 ± 2.25	15.94 ± 6.67	1.94 ± 6.16	9.46 ± 5.05

*Note*. ^*∗*^Compared with the right joint, *p* < 0.05; _ _^*∗∗*^*p* < 0.01.

**Table 5:** The unit weight torque of six joints.

	Extensor UWT			Flexor UWT		
	90°/S	180°/s	240°/s	90°/S	180°/s	240°/s
Left shoulder	16.36 ± 6.11^*∗*^	11.12 ± 6.44^*∗∗*^	6.97 ± 5.35^*∗∗*^	12.03 ± 4.34^*∗∗*^	8.77 ± 9.51^*∗*^	1.77 ± 4.48^*∗*^
Right shoulder	19.46 ± 12.39	14.41 ± 10.70	10.02 ± 8.40	14.40 ± 6.81	14.89 ± 19.30	6.03 ± 9.51
Left elbow	9.67 ± 5.09	7.50 ± 5.24	5.94 ± 4.27	9.86 ± 4.31	6.48 ± 3.68	3.42 ± 3.71
Right elbow	10.36 ± 4.34	7.49 ± 4.04	6.20 ± 3.96	11.91 ± 3.92	8.81 ± 3.68	6.09 ± 3.89
Left wrist	3.24 ± 2.27	1.37 ± 2.41	0.49 ± 1.29	3.23 ± 2.01	2.16 ± 2.24	1.39 ± 1.81
Right wrist	2.98 ± 1.88	1.25 ± 1.68	0.38 ± 1.05	3.97 ± 2.68	2.72 ± 2.53	2.38 ± 2.35
Left hip	66.65 ± 43.16^*∗∗*^	65.36 ± 108.39^*∗∗*^	52.21 ± 94.52^*∗∗*^	82.65 ± 45.67^*∗∗*^	69.37 ± 43.41^*∗∗*^	58.09 ± 38.17^*∗∗*^
Right hip	33.63 ± 18.86	26.99 ± 13.14	19.82 ± 12.48	45.17 ± 17.70	36.46 ± 16.83	30.41 ± 14.94
Left knee	61.41 ± 26.40	49.36 ± 20.57	42.85 ± 18.31	23.94 ± 9.90^*∗*^	23.95 ± 10.42	21.32 ± 9.99
Right knee	58.61 ± 15.6	47.7 ± 11.14	40.59 ± 10.9	28.17 ± 8.55	25.40 ± 7.28	22.14 ± 7.26
Left ankle	29.18 ± 8.76^*∗∗*^	25.66 ± 7.05^*∗∗*^	23.27 ± 7.49^*∗∗*^	3.73 ± 3.07^*∗∗*^	1.17 ± 1.56^*∗∗*^	0.25 ± 0.46^*∗∗*^
Right ankle	11.62 ± 11.94	6.91 ± 7.24	1.82 ± 6.20	21.60 ± 21.27	15.78 ± 14.56	13.20 ± 15.70

*Note*. ^*∗*^Compared with the right joint, *p* < 0.05; ^*∗∗*^*p* < 0.01.

**Table 6:** Correlation between indicators of SMST.

	Hand-grip	Knee bent push-up	Back muscle strength	Sit-ups	Leg muscle strength	Standing long jump
Hand-grip	1		0.596^*∗∗*^	0.234^*∗∗*^	0.504^*∗∗*^	0.398^*∗∗*^
Knee bent push-up	0.766^*∗∗*^	1	0.607^*∗∗*^	0.423^*∗∗*^	0.504^*∗∗*^	0.398^*∗∗*^
Back muscle strength	0.596^*∗∗*^		1	0.378	0.725^*∗∗*^	0.096
Sit-ups	0.234^*∗∗*^		0.378	1	0.463^*∗*^	0.367^*∗*^
Leg muscle strength	0.504^*∗∗*^		0.725^*∗∗*^	0.463^*∗*^	1	0.151
Standing long jump	0.398^*∗∗*^		0.096	0.367^*∗*^	0.151	1

^*∗*^ *p* < 0.05 (two-tailed); ^*∗∗*^*p* < 0.01 (two-tailed).

**Table 7:** Factor analysis of IST among urban boys.

*x*_1_	*x*_2_	*x*_3_	*x*_4_	*x*_5_	*x*_6_	*x*_7_
.716	−.032	.020	−.227	.199	−.484	.202
.692	.078	.127	−.193	.164	−.562	.260
.761	−.426	.023	−.086	.028	.221	.010
.678	−.458	−.070	.005	−.101	.111	−.091
.676	−.246	−.021	.493	.342	.002	−.115
.716	−.446	.052	.235	.389	.038	−.018
.326	.513	−.476	−.400	.131	.393	.142
.450	.567	−.437	−.300	−.032	.323	.157
−.052	−.462	−.083	.204	.114	.303	.072
.649	−.200	−.518	.214	.346	−.036	−.122
.432	−.648	.087	−.013	−.075	.003	.487
.570	−.263	.171	−.320	−.283	.466	.194
.081	.510	.729	.104	.119	.193	.178
.102	.356	.731	.364	.001	.059	.339
.069	.479	.207	−.001	.446	.116	−.445
.230	.728	.202	.204	.306	.076	.133
.528	.133	.279	−.600	−.017	−.119	−.311
.728	.088	.320	−.348	−.017	.008	−.393
.701	.500	−.313	.083	−.069	−.084	.156
.730	.474	−.012	.028	−.309	.041	.025
.446	−.303	.417	.253	−.097	.394	−.197
.460	−.091	.223	.008	−.667	−.247	−.206
.301	.406	−.374	.540	−.376	.035	−.041
.470	.316	−.144	.640	−.265	−.152	−.142

*Urban Boy Group*. *F*_total_ = 0.8611*x*1 + 0.6455*x*2 + 0.5112*x*3 + 0.3843*x*4 + 0.1633*x*5 + 0.0854*x*6 + 0.0799*x*7.

**Table 8:** Factor analysis of IST among urban girls.

*x*_1_	*x*_2_	*x*_3_	*x*_4_	*x*_5_	*x*_6_
.894	−.029	.046	.013	.180	−.255
.870	−.116	−.034	−.256	.089	−.196
.613	−.293	−.628	.065	−.134	.093
.410	.085	−.363	−.580	.024	.162
.150	.766	−.017	.142	.327	.273
.081	.637	.404	−.353	.321	.175
.897	−.056	−.158	.042	.084	.101
.767	.363	−.173	−.232	.167	−.107
.745	.141	.199	−.380	−.046	.097
.325	.662	.242	−.053	−.222	.185
.668	−.375	−.111	.544	.108	−.031
.666	−.435	.142	.175	.139	.363
−.089	.708	.239	.205	−.154	−.166
.494	.316	.225	.095	−.418	−.042
−.064	.436	−.600	.435	.167	.000
−.214	.317	−.420	.295	.624	−.109
−.055	−.083	.861	.046	.151	−.316
−.059	−.211	.710	−.309	.445	.000
.822	−.332	.135	−.064	.035	.083
.817	.010	−.019	.126	−.018	−.326
−.200	−.387	.115	−.010	.002	.791
−.056	−.569	.494	.371	.233	−.107
.421	.335	.346	.539	.175	.339
.412	.238	.485	.398	−.497	.076

*Urban Girl Group*. *F*_total_ = 0.7748*x*1 + 0.6637*x*2 + 0.534*x*3 + 0.1778*x*4 + 0.098*x*5 + 0.087*x*6.

**Table 9:** Factor analysis of IST among rural boys.

*x*_1_	*x*_2_	*x*_3_	*x*_4_	*x*_5_
.769	−.247	.005	.317	−.175
.792	−.224	−.198	.418	−.257
−.219	.746	−.072	.245	−.118
.870	−.094	−.206	.073	−.368
.389	.712	−.189	−.009	−.164
.400	.786	−.242	−.308	−.092
.340	.641	−.097	.660	.042
.753	.441	−.061	.230	.097
.711	.039	.095	.049	.150
.688	.289	−.391	−.181	.199
−.553	.338	.093	−.015	.512
−.444	.515	−.465	−.237	−.014
.895	−.036	−.062	−.127	−.089
.769	.064	−.293	−.290	.115
.946	−.179	.032	.047	−.084
.906	−.217	.022	.025	.096
.870	−.112	−.104	.002	−.030
.856	−.113	.210	−.317	.090
.516	.372	.627	−.240	−.005
.785	.028	−.004	−.279	.299
.252	.278	.897	.024	−.099
.288	.306	.889	−.005	−.090
.859	−.162	−.071	−.198	.248
.319	−.096	.042	.523	.684

*Rural Boy Group*. *F*_total_ = 0.8327*x*1 + 0.731*x*2 + 0.4425*x*3 + 0.1387*x*4 + 0.0944*x*5.

**Table 10:** Factor analysis of IST among rural girls.

*x*_1_	*x*_2_	*x*_3_	*x*_4_	*x*_5_	*x*_6_
.688	.352	−.083	.096	−.301	−.105
.733	.352	.104	−.176	.087	−.411
.856	−.167	−.204	.208	.006	−.123
.709	−.405	−.357	−.210	−.085	.019
.479	−.478	−.376	−.468	−.025	.254
.721	.101	−.470	.200	.318	−.100
.562	.302	−.362	.543	−.259	−.054
.530	.365	.566	−.072	−.109	.410
.545	.574	.130	−.027	.253	.204
−.043	.528	.660	.227	−.017	−.173
−.039	.145	.182	.358	.799	.283
.445	−.638	.352	.448	.033	−.124
.464	−.662	.260	.479	−.028	−.092
.877	−.369	−.011	.015	.002	−.096
.919	−.239	−.094	.040	−.017	.079
.767	.142	−.093	.267	−.132	.050
.853	.282	.085	−.019	.203	−.097
.476	−.442	.662	−.246	−.124	−.070
.664	−.374	.577	−.255	−.021	−.007
.802	.388	.147	−.092	−.079	.054
.623	.237	.052	−.517	−.025	.047
.634	−.269	−.143	−.157	.559	.179
.374	.072	−.051	.299	−.427	.671
.672	.547	−.187	−.154	−.037	−.187

*Rural Girl Group*. *F*_total_ = 0.7133*x*1 + 0.6207*x*2 + 0.3345*x*3 + 0.1159*x*4 + 0.0709*x*5.

**Table 11:** Results of the correlation analyses of SMST indicators and *F*_total_.

	AC		UB		UG		RB		RG	
	*r*	*p*	*r*	*p*	*r*	*p*	*r*	*p*	*r*	*p*
Hand-grip	0.455	<0.01	0.411	<0.01	0.507	<0.01	0.435	<0.01	0.389	<0.01
Knee bent push-ups	0.608	<0.01	0.678	<0.01	0.691	<0.01	0.522	<0.01	0.539	<0.01
Back muscle strength	0.80	<0.01	0.774	<0.01	0.803	<0.01	0.824	<0.01	0.799	<0.01
Leg muscle strength	0.811	<0.01	0.811	<0.01	0.789	<0.01	0.837	<0.01	0.801	<0.01
Standing long jump	0.465	<0.01	0.438	<0.01	0.367	<0.01	0.567	<0.01	0.478	<0.01
Sit-ups	0.425	<0.01	0.345	<0.01	0.428	<0.01	0.389	<0.01	0.516	<0.01

*Note*. AC: all children; UB: urban boys; RB: rural boys; UG: urban girls; RG: rural girls.
